# Genome of the Root-Associated Plant Growth-Promoting Bacterium *Variovorax paradoxus* Strain EPS

**DOI:** 10.1128/genomeA.00843-13

**Published:** 2013-10-24

**Authors:** Jong-In Han, Jim C. Spain, Jared R. Leadbetter, Galina Ovchinnikova, Lynne A. Goodwin, Cliff S. Han, Tanja Woyke, Karen W. Davenport, Paul M. Orwin

**Affiliations:** Department of Civil and Environmental Engineering, KAIST, Daejeon, Republic of Koreaa; School of Civil and Environmental Engineering, Georgia Institute of Technology, Atlanta, Georgia, USAb; Divisions of Biology and Environmental Science and Engineering, California Institute of Technology, Pasadena, California, USAc; DOE Joint Genome Institute, Walnut Creek, California, USAd; Los Alamos National Laboratory, Los Alamos, New Mexico, USAe; Department of Biology, California State University at San Bernardino, San Bernardino, California, USAf

## Abstract

*Variovorax paradoxus* is a ubiquitous betaproteobacterium involved in plant growth promotion, the degradation of xenobiotics, and quorum-quenching activity. The genome of *V. paradoxus* strain EPS consists of a single circular chromosome of 6,550,056 bp, with a 66.48% G+C content.

## GENOME ANNOUNCEMENT

Here, we report the finished genome sequence of *Variovorax paradoxus* strain EPS. *V. paradoxus* EPS was cultured on low-nutrient agar (5 mg/liter yeast extract [YE]) from the rhizosphere community of the sunflower (*Helianthus annuus*) on the campus of California State University, San Bernardino, CA. Other bacterial strains from this species have been identified in many different environments, including diverse soils, endophytic growth (1), and the human oral microbiota (2). Strains within this species have been shown to degrade acyl-homoserine lactone signaling compounds (3), pesticides (4), and nitrotyrosines (5), and they promote plant growth in several crops (6, 7). The genome of the potato endophyte strain *V. paradoxus* S110 was previously reported, consisting of two chromosomes with a total genome size of 6.7 Mb (1). The surface attachment and motility characteristics of *V. paradoxus* EPS have also been evaluated previously (8, 9).

The genome of *V. paradoxus* EPS was sequenced using high-throughput sequencing approaches (Illumina and 454, 30× genome coverage) from pure culture-derived genomic DNA (Promega). Sequencing and annotation were performed at the Joint Genome Institute Oak Ridge National Laboratory (JGI-ORNL) and JGI Production Genomics Facility (JGI-PGF), and finishing was done by the JGI Los Alamos National Laboratory (JGI-LANL). The finished genome was assembled using Newbler version 2.3. The genome is 6,550,056 bases in length, is organized into a single circular chromosome, and has a G+C content of 66.48%. A total of 6,088 genes were annotated, including 6,020 putative protein-coding sequences (91.25% of total bases). Of these, functions have been predicted for 73.92% (4,499 loci). A gene for 1-aminocyclopropane-1-carboxylate (ACC) deaminase was identified (Varpa_5820), consistent with a role as a plant-growth promoting rhizobacterium, along with a gene for acyl-homoserine lactone acylase activity (Varpa_4314) described previously in other *V. paradoxus* strains (1, 3). Although this bacterium is motile using a single polar flagellum, the flagellar locus was not clearly identified in this chromosomal sequence, as it was in *V. paradoxus* strain S110. Putative pilus loci were identified, as were a number of secretion systems. Two potential prophage elements (one potentially complete prophage) were also identified with the PHAST tool (http://phast.wishartlab.com [10]) in positions 1534102 to 1541586 and 2169456 to 2239232. An additional potential prophage region starting at tRNA-Lys (position 2933124, with several phage-related genes between open reading frames [ORFs] Varpa_2722 to Varpa_2770) was identified by inspection and subsequent BLAST analysis. The annotated genome data are available in IMG (see http://img.jgi.doe.gov, taxon ID 649633106), as well as NCBI (taxon ID595537) and the Genomes OnLine Database (GOLD card no. Gc01580).

### Nucleotide sequence accession number.

The finished and annotated genome sequence has been deposited in GenBank under the accession no. CP002417.
